# A novel dataset on horizontal property rights in 126 jurisdictions

**DOI:** 10.1016/j.dib.2017.03.005

**Published:** 2017-03-12

**Authors:** Giuseppe Dari-Mattiacci, Carmine Guerriero

**Affiliations:** a University of Amsterdam, The Netherlands; b University of Bologna, Italy

**Keywords:** Comparative variation, Property rights, Markets, Private takings, Predation

## Abstract

The law and the economy are deeply influenced by horizontal property rights, which are the rules regulating legal direct and indirect takings between private parties. To foster research on the determinants and impact of these institutions, we illustrate here a novel data set partially employed in (Dari-Mattiacci, Giuseppe, Carmine Guerriero, 2015; Dari-Mattiacci, Giuseppe, Carmine Guerriero, Zhenxing Huang, 2016) [3], [4], and (Guerriero Carmine, 2016) [6] and describing the acquisition of ownership through adverse possession of personal and real property and the use of government takings to transfer real property from a private party to another private party in 126 jurisdictions. These data are based on the laws and judicial decisions prevailing in each jurisdiction between 1981 and 2011.

**Specifications Table**

**Table:** 

Subject area	*Economics.*
More specific subject area	*Law and Economics; Institutional Economics.*
Type of data	*Excel.*
How data was acquired	*By extracting from questionnaires drafted by experts the rules on the acquisition of ownership through adverse possession and on government takings.*
Data format	*Raw.*
Experimental factors	*The sample has been selected based on the availability of the contributors.*
Experimental features	*Property rights are measured through indicators grounded on the laws and judicial decisions prevailing in 126 jurisdictions between 1981 and 2011.*
Data source location	*126 jurisdictions.*
Data accessibility	*Data are with this article.*

**Value of the data**

• The data can help understand the origins and impact of horizontal property rights.
• Since the private-taking-based measures of property rights we illustrate do not correlate with the most used predation-based proxies for property rights (see [6]), further research on the links among horizontal property rights, vertical property rights, and the economy is welcome.
• The data are key to draw policy implications relevant for the current process of international legal harmonization.

## 1 Data

The data set, which is reported in the “HPR_W” excel file, consists of cross-sectional observations on thirteen measures of horizontal property rights. These legal rules determine the protection of private property from private takings. These are omnipresent forms of legal expropriation, and therefore they are substantially different from predation by the state and powerful elites.^1^

## 2 Experimental design, materials and methods

We constructed each measure as follows [3,^4^,6]. First, we sent questionnaires to members of the law firms part of the international networks Lex Mundi and HG.org, participants in the World Bank doing business project, and legal scholars asking information on horizontal property rights in their jurisdiction. These contributors, whose names and affiliations are listed in the Internet appendix of [6],^2^ based their answers on the laws and judicial decisions prevailing between 1981 and 2011. Second, we extracted from these answers discrete variables assuming values higher the higher is the protection afforded to the original owner of personal or real property—i.e., “movable” or “immovable” goods in civil law parlance, vis-à-vis a good-faith possessor or a good-faith buyer from a dishonest intermediary. Because there was no relevant reform, the data take the form of a cross section of 126 jurisdictions (see Table 1).

When transaction costs are sizable, private parties are often allowed to take property with or without paying compensation to the original owner (Bouckaert and De Geest [2]). Two are the main instances in which this legal private expropriation takes place, a direct one—known as adverse possession—and an indirect one, i.e., government takings. Next, we illustrate our proxies for each (Table 2).

### 2.1 Direct private takings: Adverse possession

Adverse possession is the acquisition of property rights by a possessor by virtue of the passing of time and recurring other conditions such as continuous, notorious, and open possession. Through adverse possession then, property can be effectively transferred without the original owner׳s consent.

#### 2.1.1 Personal property

Different jurisdictions weigh differently the interests of the original owner and the possessor. In the United States for instance, a good-faith possessor of a movable good cannot acquire ownership of the good through adverse possession, whereas she/he does so after 10 years in Germany and 3 years in France. Hence, an original owner is protected increasingly more in France, Germany, and the United States, and a natural metrics of her/his property rights on a movable good is the number of years after which a good-faith possessor of the good acquires ownership or thirty, which is the maximum length of adverse possession in the sample, if she/he cannot acquire property rights, i.e., *Adverse-Possession-M-GF*. We develop in a similar way other ten variables assuming higher values the higher is the relative protection afforded to the original owner vis-à-vis the good-faith possessor/buyer of a movable good.

The first one is the number of years after which the proprietary remedies of the owner of a movable good expire, i.e., *Remedy-M*. A second set of variables refers to the purchase of a stolen movable good. These indicators gauge indeed the number of years after which the buyer acquires ownership of a stolen movable purchased within either a private sale, public market, and an auction or from a professional seller, i.e., respectively *Property-Private-Sale-SM*, *Property-Public-Market-SM*, *Property-Auction-SM*, and *Property-Professional-Seller-SM*. A third set of variables deals which the purchase of a movable good that was embezzled, i.e., withheld by someone to whom it was entrusted to be held or used for specific purposes. These indicators gauge indeed the number of years after which the buyer acquires ownership of an embezzled movable purchased within either a private sale, public market, and an auction or from a professional seller, i.e., respectively *Property-Private-Sale-EM*, *Property-Public-Market-EM*, *Property-Auction-EM*, and *Property-Professional-Seller-EM*. Finally, the dummy *Good-Faith-M* equals 0 when the good faith is presumed. Clearly, the mere presumption protects more the buyer.

#### 2.1.2 Real property

The transfer of real property is more complex since it requires registration, which has an asymmetric impact across jurisdictions depending on the extent of enforceability against third parties (Bouckaert and De Geest [2]). Accordingly, we focus on the case emphasizing the most the adverse possessor׳s claim. To illustrate, we study the conflict between a good-faith possessor without title of an immovable good and a third party buying the good from the original owner after the term for adverse possession is expired, and we maintain that the good is registered if this step is necessary to acquire property via adverse possession. Then, our proxy for horizontal property rights on immovable goods is *Adverse-Possession-I-GF*, which is defined as the number of years necessary for adverse possession of an immovable good with good-faith but without title if the adverse possessor prevails against a good-faith buyer from the original owner and/or registration is compulsory for adverse possession and thirty otherwise. When the good-faith possessor prevails, the legal protection of the original owner is weaker.

### 2.2 Indirect private takings: Government takings

Private takings can also be mediated by the state, which can always, under the power of eminent domain, seize private real property for ``public interest” and upon paying a “just” compensation [5]. What constitutes public interest however differs among different jurisdictions. With this remark in mind, we consider the dummy *Government-Takings*, which equals 1 if the state can take real property from a private party only for public use. Whenever *Government-Takings* is instead zero, the state can use government takings to transfer property from a private party to another for private for-profit use and so favor a subgroup of the population to the detriment of another.

## Figures and Tables

**Table 1:** List of Jurisdictions.

Albania; Angola; Argentina; Armenia; Australia; Austria; Azerbaijan; Bahrain; Bangladesh; Belarus; Belgium; Belize; Bolivia; Bosnia and Herzegovina; Brazil; Bulgaria; Burkina Faso; Burundi; Cameroon; Chile; China; Colombia; Costa Rica; Cóte d׳Ivoire; Croatia; Cyprus; Czech Republic; Denmark; Dominican Republic; Ecuador; Egypt; El Salvador; Estonia; Ethiopia; Finland; France; Georgia; Germany; Ghana; Great Britain; Greece; Guatemala; Honduras; Hong Kong; Hungary; India; Indonesia; Iran; Ireland; Israel; Italy; Jamaica; Japan; Jordan; Kazakhstan; Kenya; Kosovo; Kuwait; Kyrgyz Republic; Latvia; Lesotho; Lithuania; Luxembourg; Macedonia; Malaysia; Mali; Malta; Mauritius; Mexico; Moldova; Mongolia; Montenegro; Morocco; Mozambique; Myanmar; Namibia; Netherlands; New Zealand; Nicaragua; Niger; Nigeria; Northern Ireland; Norway; Oman; Pakistan; Panama; Paraguay; Peru; Philippines; Poland; Portugal; Puerto Rico; Romania; Russia; Rwanda; Saudi Arabia; Scotland; Senegal; Serbia; Sierra Leone; Singapore; Slovak Republic; Slovenia; South Africa; South Korea; Spain; Sri Lanka; Sweden; Switzerland; Syria; Taiwan; Tanzania; Thailand; Trinidad and Tobago; Tunisia; Turkey; Uganda; Ukraine; United Arab Emirates; United States; Uruguay; Venezuela; Vietnam; Zaire; Zambia; Zimbabwe.

**Table 2:** Summary of Variables.

Variables	Definition and sources	Summary statistics
*Adverse-Possession-M-GF*:	Time limit for adverse possession of a movable good by any good-faith possessor in years.	11.306 (11.890) [126]
*Remedy-M*:	Number of years after which the proprietary remedies of the owner of a movable good expire.	15.341 (12.538) [126]
*Property-Private-Sale-SM*:	Number of years after which the good-faith buyer definitively acquires property of a stolen movable good purchased within a private sale.	12.056 (12.765) [126]
*Property-Public-Market-SM*:	Number of years after which the good-faith buyer definitively acquires property of a stolen movable good purchased within a public market.	10.119 (12.332) [126]
*Property-Auction-SM*:	Number of years after which the good-faith buyer definitively acquires property of a stolen movable good purchased within an auction sale.	7.968 (11.496) [126]
*Property-Professional-Seller-SM*:	Number of years after which the good-faith buyer definitively acquires property of a stolen movable good purchased from a professional seller.	9.159 (11.911) [126]
*Property-Private-Sale-EM*:	Number of years after which the good-faith buyer definitively acquires property of an embezzled movable good purchased within a private sale.	7.782 (11.877) [126]
*Property-Public-Market-EM*:	Number of years after which the good-faith buyer definitively acquires property of an embezzled movable good purchased within a public market.	6.766 (11.267) [126]
*Property-Auction-EM*:	Number of years after which the good-faith buyer definitively acquires property of an embezzled movable good purchased within an auction sale.	5.790 (10.506) [126]
*Property-Professional-Seller-EM*:	Number of years after which the good-faith buyer definitively acquires property of an embezzled movable good purchased from a professional seller.	5.869 (10.475) [126]
*Good-faith-M*:	Dummy equal to 0 when good-faith is presumed and 1 otherwise.	0.238 (0. 428) [126]
*Adverse-Possession-I-GF*:	Number of years necessary for adverse possession of an immovable good with good-faith but without title if the adverse possessor prevails against a good-faith buyer from the original owner and/or registration is compulsory for adverse possession; thirty otherwise.	24.573 (8.082) [89]
*Government-Takings*:	Dummy equal to 1 if the state can take real property from a private party only for public use, 0 otherwise.	0.716 (0. 454) [88]

## Footnotes

1 This form of expropriation is shaped instead by vertical property rights and, albeit much less common, has been the focus of the extant social science literature [1].

2 This is available at https://sites.google.com/site/carmineguerrieroshomepage/home/research-1.
